# Detecting recurrent gene mutation in interaction network context using multi-scale graph diffusion

**DOI:** 10.1186/1471-2105-14-29

**Published:** 2013-01-23

**Authors:** Sepideh Babaei, Marc Hulsman, Marcel Reinders, Jeroen de Ridder

**Affiliations:** 1 Delft Bioinformatics Lab, Delft University of Technology, Delft, The Netherlands; 2, Netherlands Bioinformatics Centre, The Netherlands

## Abstract

**Background:**

Delineating the molecular drivers of cancer, i.e. determining cancer genes and the pathways which they deregulate, is an important challenge in cancer research. In this study, we aim to identify pathways of frequently mutated genes by exploiting their network neighborhood encoded in the protein-protein interaction network. To this end, we introduce a multi-scale diffusion kernel and apply it to a large collection of murine retroviral insertional mutagenesis data. The diffusion strength plays the role of scale parameter, determining the size of the network neighborhood that is taken into account. As a result, in addition to detecting genes with frequent mutations in their *genomic* vicinity, we find genes that harbor frequent mutations in their *interaction network context*.

**Results:**

We identify densely connected components of known and putatively novel cancer genes and demonstrate that they are strongly enriched for cancer related pathways across the diffusion scales. Moreover, the mutations in the clusters exhibit a significant pattern of mutual exclusion, supporting the conjecture that such genes are functionally linked. Using multi-scale diffusion kernel, various infrequently mutated genes are found to harbor significant numbers of mutations in their interaction network neighborhood. Many of them are well-known cancer genes.

**Conclusions:**

The results demonstrate the importance of defining recurrent mutations while taking into account the interaction network context. Importantly, the putative cancer genes and networks detected in this study are found to be significant at different diffusion scales, confirming the necessity of a multi-scale analysis.

## Background

Cancer is a complex genetic disease, caused by a multistep process in which several collaborating mutations allow a cell to evade the checkpoints that normally safeguard it against DNA damage and other disruptions of healthy cell growth [1-3]. Among all human genes more than 1% are implicated in cancer by somatic or germline mutations [4]. This implies that hundreds of genes may contribute to tumor formation. This percentage is expected to increase since cancer genome sequencing of human tumors [5] and forward genetics screens in model organisms [6-8] are providing evidence for thousands more [9]. Arguably, cancer genes are thus better considered as contributing to cancer rather than causal for cancer [10], as their effect is dependent on many other genetic aberrations.

One of the major challenges in cancer research is to determine all genes that contribute to the disease as well as delineate the pathways through which these genes exert their malicious effects on a cellular process. A potent way of discovering genes driving carcinogenesis is to perform forward genetic screens in animal models such as retroviral or transposon insertional mutagenesis (IM). Retroviral insertional mutagenesis (RIM) is based on integration of a retrovirus into the host cell’s genome, mutating the host cell’s DNA in the process. Putative cancer genes in the RIM datasets are determined by searching for genes in the vicinity of genomic regions that harbor recurrent mutations across multiple independent tumors [6,7,11]. Such regions in the genome are called Common Insertion Sites (CIS). Statistical significance of CIS can be determined by means of permutation strategies [12].

A major problem in this approach is that, assuming realistic sample sizes, not all cancer-associated genes harbor sufficient mutations in multiple tumors to be called significant. This is because mutations in the vicinity of *different* genes can exert the same deleterious effect on the *same* pathway [10]. This explains that mutations of different genes represent alternative routes to the acquisition of the same cancer hallmark [13,14]. A compelling example is given by the *Rb* pathway. Mutations in four genes *RB1*, *CDKN2A*, *CDK4*, and *CCND1* can be considered equivalent because their effect on controlling transition to replication phase is similar [10]. On the other hand, acquisitions of some cancer hallmarks require mutations of multiple genes. Examples of this include the frequently observed co-mutation of *RAS* and *TP53*[15]. Co-mutated genes can thus be considered to collaborate in carcinogenesis [16].

An important implication of this fact is that the definition of a recurrent gene mutation, defined as a gene that harbors frequent mutations in its genomic vicinity in more tumors than expected by chance, is no longer appropriate. Instead, recurrent mutations should be defined at the scale of a pathway, rather than the scale of a single gene.

A straightforward approach would be to regard mutations affecting different genes in the same pathway as recurrent by a summarization across pathways defined in databases such as KEGG [17] and using enrichment statistics such as GSEA [18]. Here, we argue against such an approach for the following reasons. First, such approaches consider a pathway as a group of genes, and thus, disregard any topological information in the gene interaction network that describes a pathway. Edge weights, capturing confidence in a certain relation, are also not taken into account. Most importantly, however, gene sets that are defined based on a pathway represent only one of many scales at which this pathway can be defined. In reality, mutations may target very specific components of a pathway (i.e. small-scale), whereas in other cases summarization across a collection of functionally similar pathways (i.e. large-scale) may be justified.

When a pathway is defined at a scale which is too small, mutations are split across different distinct pathways. As a result, mutations are only recurrent across a relatively small number of tumors. This implies loss of power to detect relevant mutation patterns. On the other hand, when a pathway is defined at a scale which is too large, many irrelevant genes are considered that do not harbor mutation. This also implies reduced power to detect recurrence. Since it is a priori unknown at which scale it is most appropriate to summarize mutations and define a pattern of recurrent mutation, a *multi-scale* approach that evaluates pathway descriptions of different scales is required.

For this reason, we propose to consider gene mutations in the context of its pathway neighborhood as encoded in a functional protein-protein interaction (PPI) network [19]. This network describes a variety of gene and protein interactions (including experimentally verified physical interactions, co-expressed genes, interactions mined from literature, known pathway interactions, interactions predicted from protein homology, etc.). Importantly, this offers the possibility to obtain a more continuous definition of the size of the pathway neighborhood, or, in other words, a less discrete definition of the *scale* at which the *interaction network context* is considered. Using a multi-scale diffusion kernel [20] (detailed in the Methods section), this scale can be varied across a range of values. This enables analysis of the mutations in interaction contexts of different scale, ranging from small-scale signaling cascades to large-scale molecular pathways.

The PPI network has been successfully used to define subnetworks with predictive value for patient prognosis from gene expression data [21-26]. Other approaches use PPI information to identify subnetworks with frequent mutations, since these may point to commonly mutated pathways [27,28]. In both cases, a scoring function is optimized to identify high-scoring subnetworks. However, these types of optimizations are NP-hard and therefore require heuristic strategies that are computationally expensive [25-27].

**Figure 1:**
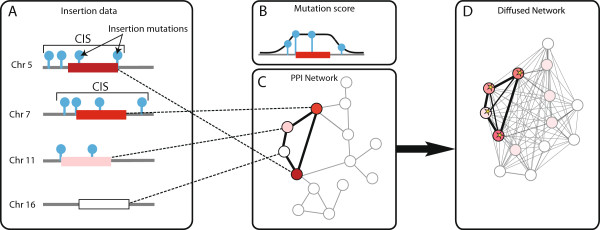
**Framework. A)** Insertion mutation data (blue lollipops, each occurring in one tumor) across a set of tumors (not shown) and four genomic regions. The region on Chr 5 and Chr 7 harbor sufficient mutations to be called CIS. The region on Chr 11 and Chr 16 do not. **B)** Gene mutation scores are obtained by weighted summarization. The weighing function is a flat-top Gaussian. **C)** PPI network with genes as nodes. Color denotes the mutation gene score. **D)** Illustration of the diffusion kernel. Conceptually, the diffusion kernel is similar to a heat diffusion process. In graphs this means that the mutation score at the graph node is diffused throughout the network dependent on the graph topology that connects this node to the rest of the network. Its global distance to the other nodes is thus dependent on the weight and number of paths connecting them as well as the diffusion strength. The latter parameter can be regarded as a scale parameter. For low diffusion strength, scores hardly diffuse and the interaction context of a gene is determined by itself and a few well-connected neighbors. For high diffusion strength, scores are almost fully diffused and can thus reach distant genes in the network, albeit in very small amounts. Using a permutation approach, it is possible to establish whether the diffused scores are higher than expected by chance (starred genes). Notably, genes with few or no mutations can still reach significance due to high scoring nodes in their neighborhood.

Here, we propose a different approach, based on diffusion kernel, to determine genes recurrently mutated in interaction context (ReMIC genes). ReMIC genes are detected when the gene itself or the interaction network neighborhood of the gene harbors more mutations than expected by chance (Figure 1). The amount of diffusion determines the scale of the network neighborhood that will be taken into account. A permutation analysis is employed to determine the significance of a ReMIC gene within the context of its network neighborhood at a particular scale. By varying this scale across a range of values, ReMIC genes that arise as a result of mutations in small, well-connected subnetworks (small-scale) as well as those that arise as a result of mutations in large pathway components (large-scale) can be identified.

The proposed approach establishes significance estimates *per gene*, mitigating the need of excessive correction for multiple testing that arises when testing many networks of different sizes. It also requires no prior assumptions on the size of subnetworks. However, the major benefit is that our method explicitly takes care of the scale at which a subnetwork is considered.

In the following, we outline the proposed methodology and show results on a large collection of viral mutation data from two large RIM screens [6,11]. In particular, we show that different networks of recurrent mutations arise at different scales. Many genes that do not harbor sufficient mutations to be called significant in a genome-based analysis are identified using the proposed approach. A significant portion of these genes are well-known cancer genes. In addition, we determine densely connected subnetworks of the significant genes which are highly enriched for cancer related pathways at different diffusion strength. These clusters also show significant patterns of mutual exclusion, indicating a functional relation between the mutated genes.

## Methods

### Mutation data

Retroviral insertional mutation data is acquired from Mutapedia (available on http://mutapedia.nki.nl). In total, the dataset contains 19 997 viral insertions in 933 tumors that have been produced in mice with various genetic backgrounds [11]. All insertions in this dataset are mapped to Ensembl genes by employing a flat-top Gaussian scoring function (Figure 1B). This scoring function is uniform within a gene and has Gaussian tails outside the gene. The width (standard deviation) of the Gaussian is chosen to be 25kb, based on observations made in de Jong *et al.*[29]. Insertions further than 50kb from the gene start or gene termination site are not associated to that gene. In this way, we obtain a (*t* × *g*) mutation matrix ***M*** where *t* is the number of tumors in the study, *g* is the number of genes in the PPI network and *M*_*ij*_ indicates the mutation score of gene *j* in tumor *i*. The mutation score vector *S* capturs the insertion frequency in the vicinity of each gene and is computed as: $S_{j}=\underset{\forall i}{\sum }M_{\text{ij}}$ (Figure 1).

### Interaction graph construction

A PPI graph is obtained from STRING [19]. The combined scores are used as interaction weights between proteins. To associate proteins in the PPI network to their corresponding genes, Ensembl gene IDs are mapped to protein IDs. In case of multiple proteins mapping to the same gene, interaction weights are collapsed into a single gene-to-gene interaction weight by averaging. We retain reliable interactions by removing all links with a weight below threshold *T*_*I*_. As a result, we obtain an undirected and weighted interaction network *N* = (*G*, *I*, *S*, ***W***), where *G* denotes a set of nodes representing the genes, *I* denotes a set of edges representing interaction edges between proteins associated with the Ensembl genes, vector *S* denotes mutation scores per gene, and weight matrix ***W*** denotes the interaction weights between the genes.

### Diffusion kernel

To represent the interaction network context of a gene, it is desirable to obtain a global similarity metric that captures the local topology of the network connecting two genes. To this end, we use the diffusion kernel approach proposed by Kondor and Lafferty [20].

The diffusion kernel of a weighted interaction graph *N* is described by:

$$K_{\beta }=e^{\beta L}$$

where *e*^*βL*^ denotes the matrix exponential and the *β* >= 0 is the diffusion parameter that controls the extent of diffusion (i.e. the scale). Matrix *L* is equivalent to the Laplacian matrix of the weighted graph *N*, and is defined by:

$$L_{\text{ij}}=\left\{\begin{array}{cc} -W_{\text{ij}} & \text{if}\;\;i\neq j\\ \overset{n}{\underset{l=1}{\sum }}W_{\text{il}} & \text{if}\;\;i=j \end{array}\right)$$

The Laplacian matrix *L* thus represents the graph topology of graph *N*, while the diffusion kernel *K*_*β*_ provides a smooth global similarity measure on *N*.

The diffusion parameter *β* plays an important role as it controls the extent of the diffusion. For *β* equal to 0, there is no diffusion (i.e. *K*_0_ = *I*) while for *β* approaching infinity the diffusion reaches equilibrium where all (connected) nodes receive the same diffusion contribution. Diffusion Diffusion kernel matrices are created for a range of *β* values. Finally, each diffusion kernel matrix is applied to the gene mutation score vector, to obtain diffused mutation scores, by calculating *SK*_*β*_.

### Permutation procedure

Significance estimates, establishing whether a gene’s diffusion score is higher than expected by chance, are calculated on a per *gene* and per *scale* basis by permutation analysis. To this end, we randomize the association between genes and mutations by permuting the mutation scores in each tumor independently and recalculating the overall mutation score vector *S*. The diffusion kernel at a particular scale is then applied to the randomized *S*. This procedure is repeated 10^7^ times, resulting in a null-distribution per gene from which an empirical *p*-value (including pseudocount) can be calculated.

To reduce the computational burden, we precompute 10^5^ random score vectors *S* and store these in vector *S*_rnd_. In every permutation iteration, *g* scores are randomly selected from *S*_rnd_. Moreover, the permutation procedure for a gene is discontinued when more than 10 permuted diffused scores exceed its non-permuted diffused score. As a result, the permutation procedure is procedure is carried out for fewer genes, reducing the computational burden considerably. Correction for multiple testing is achieved by using the method of Storey and Tibshirani to calculate a false discovery rate (FDR) per gene [30]. The *p*-values are adjusted by considering the number of genes in the PPI network as well as the number of scales at which the diffusion kernel is employed. Genes exceeding the *α*-level are called ReMIC genes. The source code is available in Additional file 1.

### Graph clustering

In this study, we divide subnetworks of significant genes into densely connected subgroups by applying the Girvan-Newman graph clustering method that uses a natural division of the graph into clusters based on the strength of connections between the nodes [31]. In this algorithm, a betweenness score of an edge is computed based on the number of shortest paths that flow through that edge. A high betweenness score means that this particular edge connects clusters with densely connected nodes. A dendrogram is constructed by gradually removing the edges with the highest betweenness score. The root of the dendrogram represents the original network and the leaves are individual genes (Additional file 2: Figure S6-S11). For each division of the network into clusters a *modularity* measure is calculated which indicates the fraction of edges in the network that connect nodes in the same clusters. The dendrogram is cut at the point where the maximum modularity is achieved.

To calculate a modularity measure for a particular division of the network with *n* clusters a symmetric (*n* × *n*) matrix ***E*** is defined. The matrix entry *E*_*ij*_is the fraction of all edges in the network that connect nodes in cluster *i* to nodes in cluster *j*. The trace of ***E*** (*T*_*E*_) indicates the fraction of edges in the network that link nodes in the same cluster. Accordingly, a high value of *T*_*E*_ demonstrates a good division of the network into densely connected clusters. However, *T*_*E*_ itself yields no information about the structure of clusters, since, for example, a single cluster consisting of all nodes results in a maximal value of *T*_*E*_while it is not a good division of the network. For this reason the trace of ***E*** - ∥***E***^2^∥ is used as a modularity measure where ∥***E***^2^∥ indicates the sum of the entries of the matrix ***E***^2^. This modularity measure is the fraction of the edges in the network that link nodes in the same cluster minus the expected value of the same quantity for randomized network (in which edges are distributed at random without considering the structure of clusters). A modularity measure equal to zero means that the number of within-cluster edges is not better than random connections. A good division of densely connected clusters results in a modularity measure close to one. The modularity measure is calculated for each division of the network and the best cut-level of the dendrogram is determined based on the maximum modularity. In this work, we use Cytoscape plugin [32] GLay community structure analysis that implements the Girvan-Newman algorithm [33].

### KEGG pathway enrichment

In order to get a general idea of the function of ReMIC gene clusters, KOBAS [34] is applied to calculate KEGG pathway enrichments. More specifically, we use KOBAS to determine overlap of a ReMIC gene cluster with all known pathways in the KEGG database [17]. For each pathway that occurs in the input gene set, KOBAS counts the total number of input genes that are involved in the pathway. It also counts the total number of genes in the background gene set (all genes in the interaction network) that are involved in the same pathway. A *p*-value of the pathway enrichment is calculated using the Fisher exact test with Benjamini FDR correction to adjust for multiple testing.

### Mutual exclusivity analysis

To determine whether the genes in a subnetwork exhibit more than random mutual exclusion of mutations (i.e. mutations in different genes of the subnetwork tend *not* to co-occur within the same tumor), we employ the mutual exclusivity module (MEMo) approach [35]. The mutation matrix ***M*** is considered as the adjacency matrix of a graph where *M*_*gt*_ > 0, represents an edge connecting gene *g* to tumor *t*. The switching permutation technique is applied to generate 10^4^ random mutation matrices [36].

In the switching permutation method, a randomized mutation matrix is generated from the original mutation matrix ***M*** while the overall adjacency distribution between genes and tumors is preserved. To do this, two edges are randomly selected between gene *g*_1_ and tumor *t*_1_(*g*_1_, *t*_1_) and gene *g*_2_ and tumor *t*_2_(*g*_2_, *t*_2_). If these edges appear in the adjacency graph of ***M*** (i.e. $M_{g_{1}t_{1}}$ and $M_{g_{2}t_{2}}$ are non-zero entries) the ends of the are non-zero entries) the ends of the edges are swapped to give (*g*_1_, *t*_2_) and (*g*_2_, *t*_1_). This exchange is only allowed if it generates no duplicate edges between a gene and a tumor. The process is repeated for *k*×*q* iterations where *k* is the total number of edges in the adjacency graph of ***M*** and *q* is a constant number. It has been empirically shown that *q* = 100 is adequate for many networks [36].

The resulting random mutation matrices are then used to calculate an empirical *p*-value for a gene cluster. The *p*-values are calculated as the fraction of permutations that lead to a greater or equal number of mutations than those observed in ***M***. Genes within significant clusters (with *α*-level of 0.05) are considered to be more mutually exclusive than expected by chance.

## Results and discussion

### Identifying Genes Recurrently Mutated in Interaction Context (ReMIC)

We apply our method to the obtained interaction graph (*N*) as described in the Methods section. This graph contains reliable interaction links with scores more than the median value of the combined scores of all interactions (*T*_*I*_ = 500). This is a trade-off between high-confidence interactions (score > 700) and low-confidence interactions (score < 400) [19]. The resulting interaction graph *N* with edge weights ***W***, contains 14 842 nodes and 448 472 edges. The mutation scores *S* are computed as described in the Methods section for all genes present in the interaction network.

The diffusion kernel is applied to the complete PPI graph *N* for a range of scales by varying *β* in the range [0, 0.03]. A permutation procedure is performed to obtain *p*-values for each gene and each scale separately. Genes recurrently mutated in their interaction context (ReMIC genes) are defined as nodes that are significant at an *α*-level of 0.001 (Additional file 3). ReMIC genes are presented within the context of their original protein-protein interactions. The resulting ReMIC network is clustered using Girvan-Newman clustering technique (Additional file 2: Figure S6-S11). The resulting clusters are called ReMIC gene clusters.

### ReMIC genes are organized in connected components

Inspection of the significant genes at the various diffusion scales reveals that the vast majority of them are organized in connected components. This demonstrates that, even though the significance estimates are performed on a per gene bases, the diffusion process captures groups of significant genes that are connected in the PPI network.

**Figure 2:**
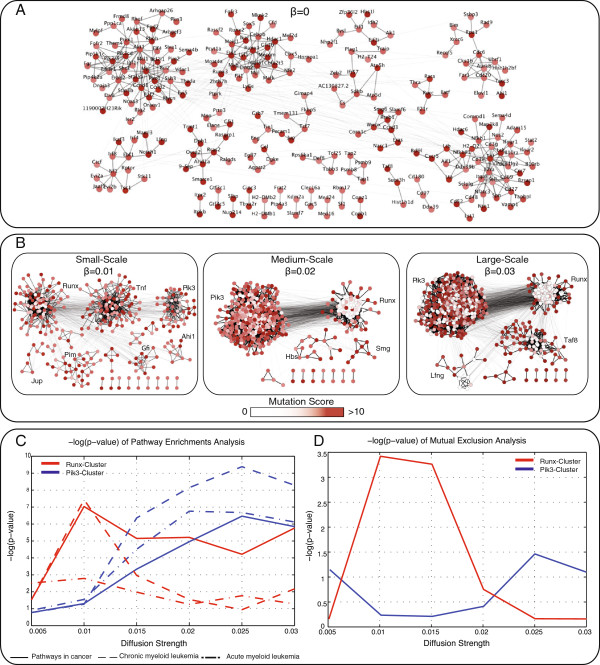
**Gene clusters across the diffusion scales. A)** The CIS genes shown in the context of the interaction network which are significant without any diffusion (*β* = 0). These genes represent the major sources of mutation score, and drive low scoring genes in the diffusion process. **B)** Overview of the identified ReMIC gene clusters at three scale levels: small-, medium- and large-scale. The color of the nodes reflects the mutation score *before* diffusion. Two major gene clusters, Runx- and Pik3-cluster are apparent across the scales. **C)***p*-values of pathway enrichment analysis of three pathways in the KEGG database for two detected clusters in all diffusion scales. Both clusters are highly enriched for *pathways in cancer* across a range of scales. The Runx- and Pik3-cluster show strongest enrichment for the small- and large-scale, respectively. **D)***p*-values of mutual exclusivity analysis for the two ReMIC gene clusters. Both clusters are significantly mutually exclusive across all scale levels. The minimum *p*-value is, however, attained for the scale at which the strongest KEGG enrichment was observed.

For *β* equal to 0 there is no diffusion. Hence, all significant genes at this scale harbor sufficient mutations to be called significant irrespective of their protein interaction context. Indeed, the collection of significant genes at this scale represents the known CIS network, since virtually all of these genes have been previously discovered in the analysis of the original screen using traditional CIS analysis [11]. A substantial fraction of them are known tumor suppressors or oncogenes such as *Gfi1*, *Notch1* and *Myc*[37]. Figure 2A shows these CISs and the protein-protein interactions between them.

When the scale is increased, genes gradually diffuse their scores across the network. High scoring nodes that are connected to many low scoring nodes diffuse their score rapidly, whereas low scoring nodes with few connections diffuse their score more slowly. As a result, genes with relatively low (pink) or zero (white) mutation score are more included at higher scales as demonstrated in Figure 2B for three specific diffusion scale levels (*β*) representing small-, medium- and large-scale. Therefore, while CIS genes have high initial scores, the ReMIC genes arise as a result of mutations in their network neighborhood rather than in their genomic vicinity alone. It should be noted that the CIS genes do contribute to the significance of ReMIC genes by virtue of score diffusion through the local graph topology.

### ReMIC gene clusters strongly enriched for cancer related pathways

A striking observation from the ReMIC gene clusters across the scales is that, in addition to some small clusters, two major gene clusters are apparent (indicated in Figure 2B). It is apparent that these clusters gradually increase size as the scale parameter increases. In the following, we refer to these clusters as the Runx- and Pik3-cluster.

**Figure 3:**
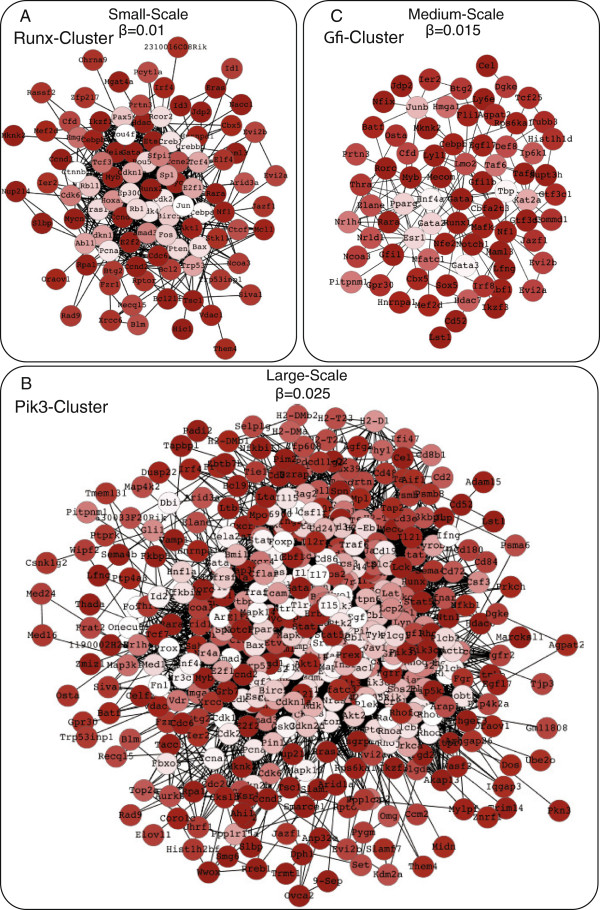
**Prominent ReMIC gene clusters.** ReMIC genes in the relevant diffusion scale of **A)** Runx-cluster (consisting of 100 ReMIC genes at the small-scale), **B)** Pik3-cluster (consisting of 436 ReMIC genes at the large-scale) and **C)** Gfi-cluster (consisting of 75 ReMIC genes at the medium-scale). The clusters are visualized using Organic Cytoscape layout [32]. The color of the nodes reflects the mutation score *before* diffusion.

To examine if one scale is more relevant than other scales, we investigate KEGG pathway enrichments of the genes in the Runx- and Pik3-cluster across different scales (Additional file 4). The results of this analysis are presented in Figure 2C, and show that both clusters are highly enriched for the general *pathways in cancer* category. It furthermore shows that the enrichment becomes more pronounced when the scale is increased and the minimum *p*-value is attained at different scales for each cluster. The highest enrichments occur at the small-scale for the Runx-cluster (Figure 3A) and the large-scale for the Pik3-cluster (Figure 3B). Similar observations can be made for leukemia pathways (dashed lines in Figure 2C). Notably, overlaps between the insertional mutations in mouse lymphomas and copy number variation in human *acute myeloid leukemia* and SNPs in human *chronic myeloid leukemia* have been observed previously [11].

In addition to the Runx- and Pik3-cluster, the Gfi-cluster is apparent at the medium-scale. This cluster includes important cancer related genes such as *Gfi1*, *Myb*[11] and *Nocht1*[10] (Figure 3C). The Gfi-cluster is enriched for *pathways in cancer* category (*p*-value = 0.06) and *chronic myeloid leukemia* pathway (*p*-value = 0.04). Interestingly, this cluster is exclusively apparent at the medium-scale.

Next, for the Runx- and Pik3-cluster, we select the scales for which the minimum *p*-value is attained and determined enrichments in other KEGG pathways. Table 1 summarizes the results by showing the *p*-value and rank of the most enriched pathways. Although most prominent cancer related pathways are enriched for both clusters, the top-ranked pathways are notably different. Taken together, these results clearly illustrate the benefit of multi-scale analysis when determining ReMIC gene clusters.

**Table 1:** Pathway enrichments

**KEGG pathways**	**Pik3-cluster**		**Runx-cluster**	
	**Rank**	***p *-value**	**Rank**	***p *-value**
Cell cycle	60	0.11	1	1.7E-10
Prostate cancer	4	1.4E-07	2	1.3E-08
Chronic myeloid leukemia	2	4.1E-10	3	3.6E-08
Pathways in cancer	8	3.4E-07	4	9.2E-08
HTLV-I infection	28	0.001	5	5.2E-07
Small cell lung cancer	20	4.6E-05	6	8.9E-07
Colorectal cancer	14	2.1E-06	7	8.3E-06
Glioma	3	3.1E-08	8	5.2E-05
p53 signaling pathway	73	0.27	9	5.3E-05
Transcriptional misregulation in cancer	77	0.36	10	5.3E-05
T cell receptor signaling pathway	1	1.1E-11	35	0.47
B cell receptor signaling pathway	5	1.5E-07	34	0.44
Non-small cell lung cancer	6	1.6E-07	13	0.0002
Fc epsilon RI signaling pathway	7	3.2E-07	65	0.95
ErbB signaling pathway	9	7.9E-07	21	0.05
Pancreatic cancer	10	8.7E-07	14	0.0003

The top ranked pathways of KEGG enrichment analysis for the Runx- and Pik3-cluster determined at the small- and large-scale, respectively (Figure 3A, B).

### ReMIC gene clusters exhibit a pattern of mutual exclusive mutation

A hallmark of cancer is that mutations of many different genes can lead to alteration of the same pathway. Moreover, once the proliferative advantaged is conferred, additional mutations in the deregulated pathway are less likely to occur. Mutually exclusive mutations, therefore, provide evidence that the altered genes are functionally linked in a common biological pathway [35]. To test this conjecture, we employ the test for mutual exclusion presented in the MEMo algorithm [35]. To this end, the MEMo permutation procedure is applied to identify whether the clustered ReMIC genes exhibit a pattern of mutual exclusion that extends beyond one expected by chance. The results are shown at Figure 2D for the Runx- and Pik3-cluster across the scales.

From these results it follows that both clusters exhibit a significant pattern of mutual exclusion (*p*<0.05). It is particularly striking that the strongest significance levels for both clusters arise at different scales. For ReMIC genes within the Runx-cluster, mutual exclusion is strongest at the small diffusion scale (Additional file 2: Figure S4), whereas for the Pik3-cluster this occurs at the large-scale (Additional file 2: Figure S5). These scales coincide with the scales of maximum functional enrichment (Figure 2D). These results illustrate the importance of a multi-scale definition of the pathway context in which mutations occur.

### ReMIC clusters include many non-CIS genes

For all ReMIC clusters with *β* > 0 it is apparent that they contain genes that harbored no or only few mutations (indicated by white or pink nodes in Figure 2B). Therefore, these genes would not have been discovered in a traditional CIS analysis.

Many of the white ReMIC genes are also known and prominent cancer genes. Examples include *Ctnnb1*, *Jun*, *Bcr* and *Src*, which are detected across all the scales. Other cancer genes are exclusively detected at larger scales, such as *Smad4* and *Ntrk1* in the Pik3-cluster. These genes harbor no mutations in the RIM dataset and are thus undetectable unless the interaction context is taken into account.

Also, many pink ReMIC genes represent well-characterized cancer genes. A prominent example is the *Rbl1* gene in the Runx-cluster. *Rbl1* is a tumor-suppressor gene found to be dysfunctional in many human tumors including leukemia [11]. Other examples include *Raf1* and *Ptk2* in the Pik3-cluster [38]. In our data, these genes are hit in only few tumors, and hence, mutation scores are insufficient to reach significance thresholds.

The question arises whether pink ReMIC genes are selected simply because they are part of the network neighborhood of important cancer genes (i.e. the CIS genes) or, more interestingly, that they are selected due to the combination of local graph topology and distribution of mutations across this topology.

To examine this hypothesis, we compare the pink ReMIC genes from the Runx- and Pik3-cluster with sets of genes that are not significant in our analysis but that are connected to one of the high-scoring genes in the same cluster. For both groups of genes, that both harbor few mutations, enrichments in the two leukemia pathways and pathways in cancer are determined. The results are summarized in Table 2. From these results it follows that pink ReMIC genes from both clusters rank higher in the enrichment test for two out of three tested relevant pathways. This is especially apparent for the larger Pik3-cluster, for which substantially lower *p*-values are obtained for enrichment in the two leukemia pathways. We therefore conclude that the local network topology plays an important role in determining whether genes are involved in deregulating a pathway.

**Table 2:** Pink ReMIC genes

**KEGG pathways**	**Pink nodes in Pik3-cluster**				**Pink nodes in Runx-cluster**			
	**Significant**		**Non-significant**		**Significant**		**Non-significant**	
	***p *-value**	**Rank**	***p *-value**	**Rank**	***p *-value**	**Rank**	***p *-value**	**Rank**
AM leukemia	0.002(17/294)	26	0.54(10/933)	61	0.01(6/81)	16	0.001(11/265)	3
CM leukemia	2.5E-07(31/294)	2	0.9(9/933)	101	4.5E-06(12/81)	3	0.001(10/265)	9
Pathways in cancer	8.7E-06(67/294)	11	0.54(45/933)	62	1.3E-05(23/81)	4	2.1E-05(34/265)	1

*p*-values of pathway enrichment for ReMIC genes and non-selected pink nodes which are connected to the red nodes in the

Runx- and Pik3-cluster. Numbers between parentheses show the number of implicated genes in that particular pathway / total

number of genes in the set.

### ReMIC genes co-localize in leukemia pathways

**Figure 4:**
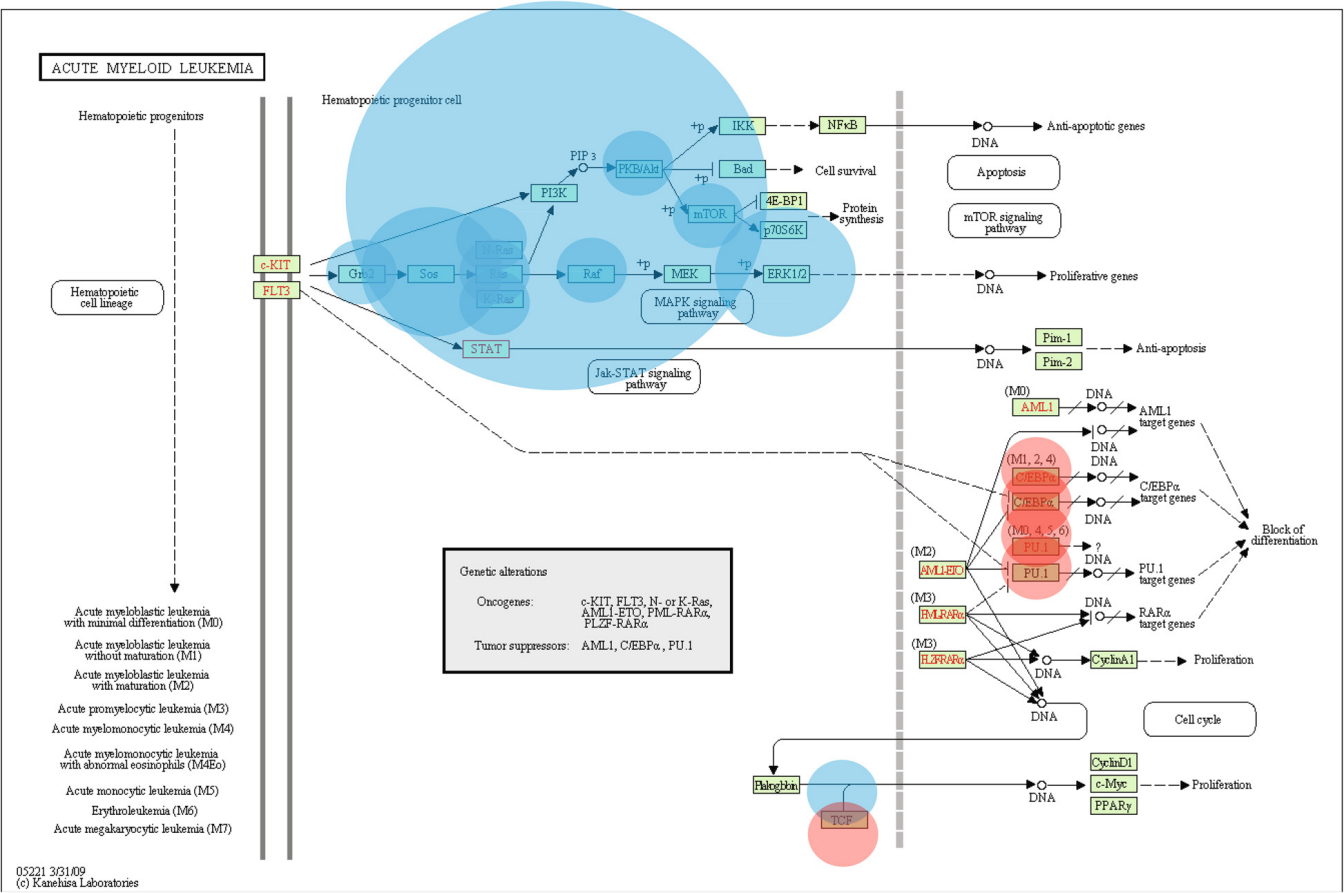
**Superimposed on the KEGG pathways.** The white and pink ReMIC genes are co-localized in a confined part of the *acute myeloid leukemia* pathway. Non-CIS ReMIC genes in the Pik3- and Runx-cluster all co-localize in the top left (blue) and in bottom right (red), respectively.

The overlaps between retroviral mutations in mouse lymphomas and human myeloid leukemia have been previously observed [11]. Therefore, most of the selected CIS genes are implicated in leukemia pathways. To identify whether the white and pink ReMIC genes are also part of these pathways we superimpose non-CIS ReMIC genes that are detected in the Runx- and Pik3-cluster at the scales for which the strongest KEGG enrichments are observed and the *acute myeloid leukemia* KEGG pathway. ReMIC genes are labeled according to their appearance in either the Runx- (red) or Pik3-cluster (blue) cluster (with circle size indicating the number of ReMIC genes within each KEGG meta-node). The result is illustrated for the *acute myeloid leukemia* pathway in Figure 4 (similar observations can be made for the *chronic myeloid leukemia* pathway, shown in the Additional file 2: Figure S1).

This reveals that a clear co-localization of the ReMIC genes in a confined part of the pathway is found. It is particularly striking that non-CIS ReMIC genes from the Pik3-cluster all co-localize in the top left of this KEGG pathway (blue circles in Figure 4). These genes play important roles in the *ErbB* and *Mtor* signaling pathways, among others. The non-CIS ReMIC genes in the Runx-cluster, on the other hand, co-localize in the bottom right. Similar observations are made in case CIS genes (red ReMIC genes) are included (shown in the Additional file 2: Figure S2 and S3).

Consequently, although this KEGG pathway is enriched for ReMIC genes, mutations appear to be clustered in clear mutation hot-spots of the pathway. Moreover, the ReMIC gene clusters comprise many other genes that are part of other pathways or not present in any of the pathways. This demonstrates the importance of a multi-scale approach that is based on protein-protein interactions as opposed to restricting the search for recurrent mutations to single genes (CIS analysis) or enrichment of whole discretely defined pathways.

## Conclusions

Recently, several methods in computational cancer biology have been developed to identify significantly mutated pathways using biological networks such as a PPI graph [21,24-27]. The main strategy of these approaches is to search for high-scoring subnetworks in terms of a specific scoring function. Due to the fact that such problems are NP-hard, greedy and heuristic approaches are employed [25-27]. In this study, we propose a graph diffusion framework to overcome this difficulty.

The multi-scale graph diffusion is applied to detect recurrent gene mutation in the context of the functional protein-protein interaction network. Using graph diffusion, it is possible to capture the local topology of the interaction network and diffuse gene mutation scores across the interaction graph. The diffusion strength acts as a scale parameter, determining the size of the network neighborhood across which recurrence is detected. Recurrently mutated genes in interaction context (ReMIC genes) are identified by permutation analysis per gene and scale. This reduces the computational complexity of examining the virtually limitless number of possible subnetworks within the complete PPI network.

We apply this method to discover recurrent gene mutations in a large collection of viral mutation data. We demonstrate that significant genes (called ReMIC genes) are organized in connected components even though the significance estimates are performed on a per gene bases. These clusters of ReMIC genes are apparent across multiple diffusion scales. In addition to some small clusters, two major gene clusters are apparent: the Runx- and Pik3-cluster. We demonstrate that these clusters are strongly enriched for cancer related pathways. Moreover, genes within these clusters are significantly mutually exclusive at particular diffusion scales, supporting their functional relation in tumorigenesis. Many ReMIC genes harbor no or infrequent mutations such as *Ctnnb1*, *Bcr*, *Src* and are, therefore, not detectable in an ordinary CIS analysis. Literature provides substantial evidence for their roles in cancer, particularly in leukemia, illustrating the efficacy of identifying cancer genes in the context of their functional interaction network.

Graph diffusion has been explored previously to identify subnetworks harboring frequent mutations in a method called HotNet [27]. One of the most important differences with our method is the way in which the significance estimates are calculated. In HotNet, an approximate solution for the connected maximum coverage problem is adopted to find significant mutated subnetworks. This solution is heuristic and computationally expensive since a myriad of subnetworks of different sizes need to be evaluated. Importantly, in order for this to become computationally tractable, white nodes need to be removed and are thereby excluded from appearing in the resulting subnetworks. In our method, on the other hand, the whole PPI network is considered.

HotNet furthermore requires the user to predefine the size of the subnetworks that it will find. Our approach, on the other hand, mitigates the need of making prior assumptions on the size of the ReMIC gene clusters. As a case in point, we analyze our insertional mutagenesis data with the HotNet algorithm and demonstrate that, at a particular diffusion scale, our method outperforms HotNet to detect distinct subnetworks that are related to known cancer pathways (see Additional file 2: Supplemental text).

Most importantly, however, we show that for different scale levels different functional enrichments, mutual exclusion patterns and ReMIC genes are observed. Moreover, for certain scale levels, clusters of ReMIC genes exhibit a clear co-localization in specific hot-spots of the KEGG representation of the leukemia pathway. Taken together, this demonstrates the importance of analyzing gene mutation in the context of their interaction network in a multi-scale fashion.

## Competing interests

The authors declare that they have no competing interests.

## Authors’ contributions

SB implemented the methodologies and analysed the data. MH contributed to design of the diffusion framework. MR and JdR conceived the experiments and mentored the project. JdR and SB wrote the manuscript with contributions from MR. All authors revised, discussed, and amended the manuscript and approved its final version.

## Supplementary Material

Additional file 1 **Source code.** A Zip file containing the source code of the presented algorithm for Matlab and R and related files. These files are also available on http://bioinformatics.tudelft.nl/users/sepideh-babaei. Note that the results of this study are calculated by Matlab. Click here for file

Additional file 2 **Supplementary text.** A PDF file containing the supplemental figures and text. It includes a figure of the ReMIC genes superimposed on the leukemia KEGG pathways, results of the analysis of the insertional mutagenesis data with the HotNet algorithm, dendrograms extracted by the graph clustering technique as well as figures of the mutation profiles of the ReMIC genes. Click here for file

Additional file 3 **Lists of ReMIC genes across the scales.** MS Excel XLS tables contain ReMIC genes which are selected across the diffusion scales. Click here for file

Additional file 4 **KEGG pathway enrichments table.** MS Excel XLS tables containing enriched KEGG pathways for both Runx- and Pik3-cluster across the diffusion scales. Click here for file
