# Supplementary material for: Detecting recurrent gene mutation in interaction network context using multi-scale graph diffusion
Source: BMC Bioinformatics. 2013 Jan 23;14:29. doi: 10.1186/1471-2105-14-29 (PMC3626877; doi:10.1186/1471-2105-14-29)
Supplement: Additional file 2 — Supplementary text. A PDF file containing the supplemental figures and text. It includes a figure of the ReMIC genes superimposed on the leukemia KEGG pathways, results of the analysis of the insertional mutagenesis data with the HotNet algorithm, dendrograms extracted by the graph clustering technique as well as figures of the mutation profiles of the ReMIC genes. [file 1471-2105-14-29-S2.pdf]

Detecting recurrent gene mutation in interaction network context using multi-scale graph diffusion

Sepideh Babaei, Marc Hulsman, Marcel Reinders, Jeroen de Ridder

Supplementary materials

ReMIC genes co-localize in leukemia pathways

We superimpose ReMIC genes that are detected in the Runx- and Pik3-cluster at the scales for which the strongest KEGG enrichments are observed and the leukemia KEGG pathways. ReMIC genes are labeled according to their appearance in either the Runx- (red) or Pik3-cluster (blue) cluster (with circle size indicating the number of ReMIC genes within each KEGG meta-node).

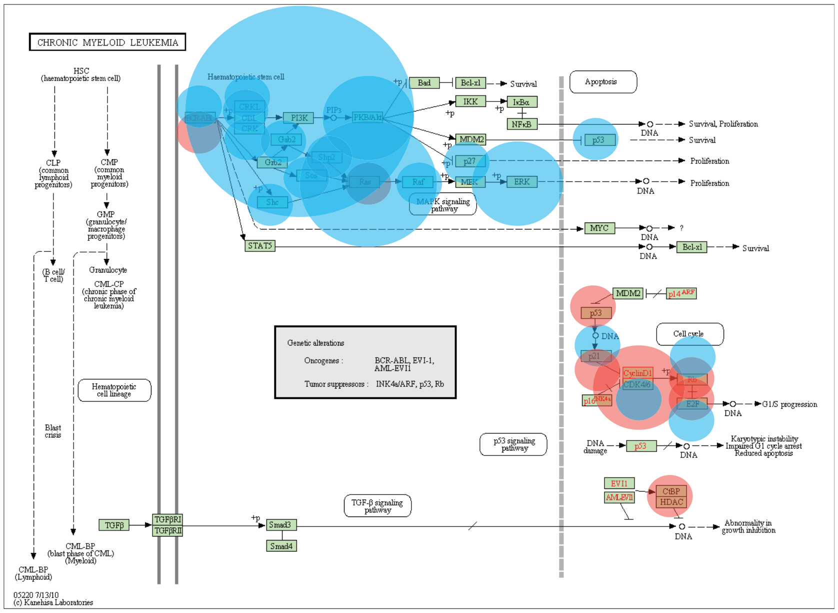

Figure S1: The white and pink ReMIC genes are co-localized in a confined part of the *chronic myeloid leukemia* pathway. Non-CIS ReMIC genes in the Pik3- and Runx-cluster all co-localize in the top left (blue) and in bottom right (red), respectively.

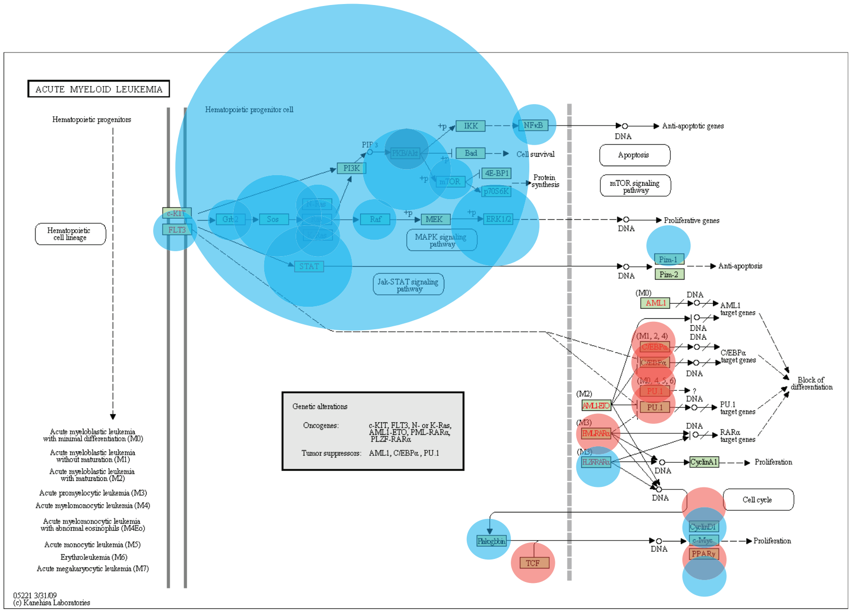

Figure S2: The ReMIC genes are co-localized in a confined part of the *acute myeloid leukemia* pathway. ReMIC genes in the Pik3- and Runx-cluster all co-localize in the top left (blue) and in bottom right (red), respectively.

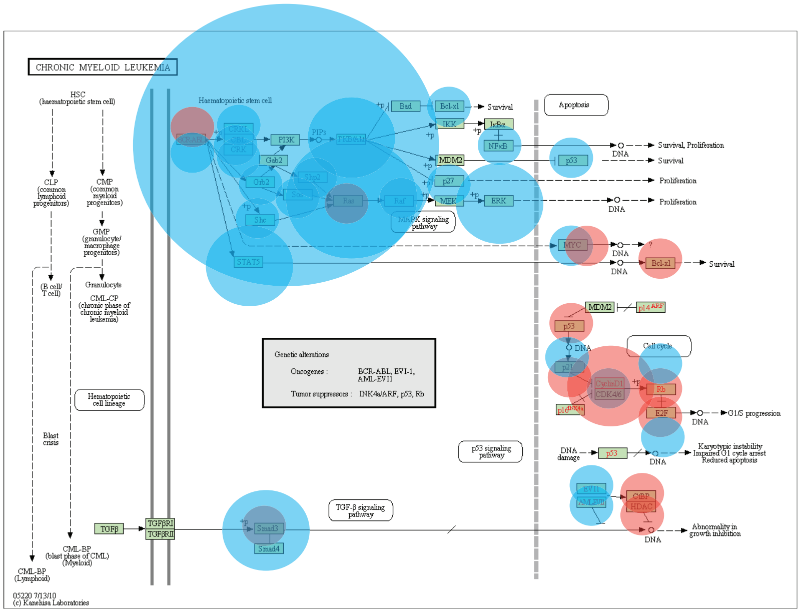

Figure S3: The ReMIC genes are co-localized in a confined part of the *chronic myeloid leukemia* pathway. ReMIC genes in the Pik3- and Runx-cluster all co-localize in the top left (blue) and in bottom right (red), respectively.

Analysis of the insertional mutagenesis data with the HotNet algorithm

We analyze our insertional mutagenesis data with the HotNet algorithm available on http://compbio.cs.brown.edu/software.html.

First, the influence graph (i.e. the kernel matrix) is calculated by applying the diffusion kernel on the PPI network. The diffusion strength is set to 0.1. To increase the computational costs, HotNet removes all edges with weight less than a threshold δ. The derived δ value is 0.01 which is selected based on the permutation analysis. Then, the reduced influence graph is captured by removing all genes that harbor mutations in more than one tumor. Thus, significant connected components are explored in the graph including 4135 genes.

The resulting connected components include several small subnetworks (with two or three genes) as well as one larger subnetwork. We identify two significant mutated subnetworks with more than 5 genes (p-value $< 10^{-3}$). The small one consists of five pink genes: *Olfr221*, *Olfr1396*, *Olfr314* and *Olfr373* mutated in three samples, and *Arrb2* mutated in four samples of our insertional mutagenesis data. The extremely large derived subnetwork includes most of the red and pink nodes in the PPI graph.

We find that the number of connected components is strongly dependent on the value of threshold δ. Thus, we increase this threshold to 0.1 to examine if we identify more connected components of the reasonable sizes. In the results, in addition to one large subnetworks with 2008 genes, we detect six mutated subnetworks of size ≥ 7. They are not enriched for the general *pathways in cancer* category nor for the leukemia pathways. The most enriched pathway and the genes apparent in each component are reported in Table S1.

Table S1: The most enriched pathway of the connected components of size ≥ 7 derived from the reduced influence graph with $\delta = 0.1$. Numbers between parentheses show the number of mutations of the genes in the samples.

size	Genes	Pathway	p-value
29	Plekhg2(4),Plekhg5(3),Arhgap30(2),Abr(4) Arhgap15(4),Rhoq(3),Rhoh(19),Arhgap9(8) Arhgap10(5),Arhgap6(4),Rhog(5),Rhof(13) Rhobtb1(2),Arhgef3(16),Rhot2(9),Hmha1(5) Obscn(2),Arhgef18(5),Syde2(3),Fgd2(38) Arhgap19(11),Arhgap22(4),Vezf1(4) Arhgap25(6),Arhgap24(2),Arhgef17(5) Arhgap18(3),Arhgap4(5),Arap1(11)	PDGF signaling pathway	0.0004
9	Mat2b(2),Srm(3),Gm853(3) Hemk1(5),Amd1(5),Ahcyl2(3) Sat2(6),Mat2a(6),Mtrf1l(3)	Methionine degradation	0.0001
7	Dolk(5),Pigl(11),Pigq(4),Pigv(16) Pigw(5),Pigx(4),Dpm3(3)	Dolichyl-diphosphooligosaccharide	0.005
15	Tssk6(3),Emd(3),4831426I19Rik(6) Tsks(4),Sun2(4),Sun1(6),Cdk17(14) Mfsd4(6),Elk3(11),Elk4(5),Serpinb5(3) Cables1(4),Cdk5r1(3),Ptprh(8),Plec(3)	Meiotic Synapsis	0.003
8	Cpm(4),Ace(3),Pgpep1(11),Camkv(13) Gng7(6),Htr1f(3),Dpp4(7),Prep(4)	5HT1 type receptor mediated	0.366
7	Nkiras1(3),Nfkbib(7),Nfkbie(4),Ppp6r3(4) Ppp6r1(8),Bcl3(6),N4bp2(10)	Toll receptor signaling pathway	0.009

ReMIC gene clusters exhibit a pattern of mutual exclusive mutation

Mutation profiles of the ReMIC genes demonstrate that high scoring genes play an essential role in the pattern of mutual exclusive mutations (Figure S4 and S5). For example, in the Pik3-cluster, *Gfi1* harbors mutations in more than 50% of the tumors (470 among all 933 tumors). The tumors without a mutation in *Gfi1* frequently harbor mutations near *Myc*, *Ccnd3* and/or *Rras2*. Moreover, *Notch1* or *Rt3* is mutated in tumors in which most of the other genes harbor no mutation. Similar observations can be made for ReMIC genes in the Runx-cluster. For example, *Myb* and *Kzf1* are mutated in different sets of tumors.

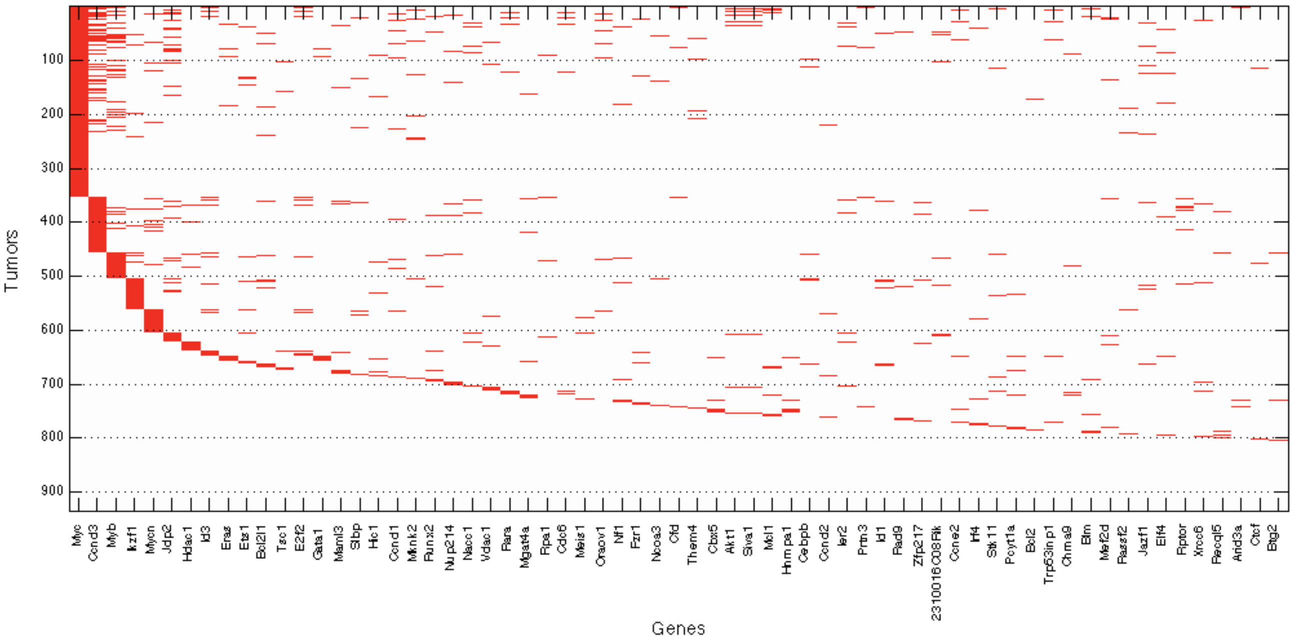

Figure S4: Mutation profile of ReMIC genes in Runx-cluster at the small-scale. For each gene (columns), tumors (rows) with or without mutations are indicated by red or white, respectively. In this plot, genes harboring mutation in more than 10 tumors are shown (65 ReMIC genes).

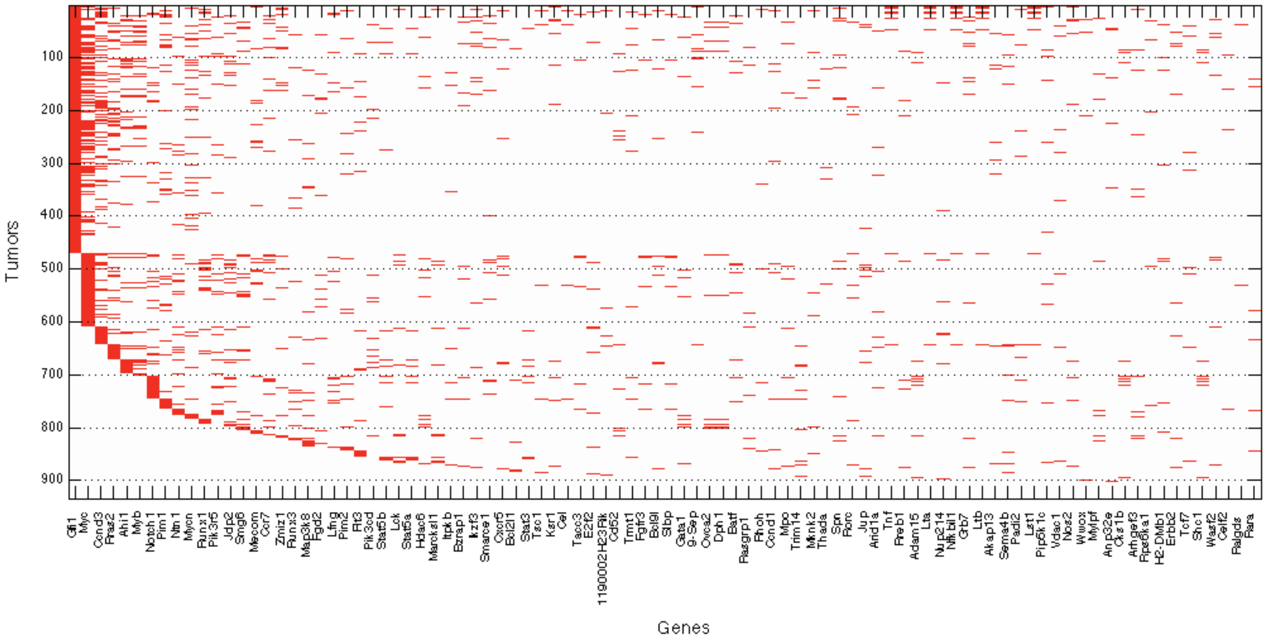

Figure S5: Mutation profile of ReMIC genes in Pik3-cluster at the large-scale. For each gene (columns), tumors (rows) with or without mutations are indicated by red or white, respectively. In this plot, genes harboring mutation in more than 15 tumors are shown (92 ReMIC genes).

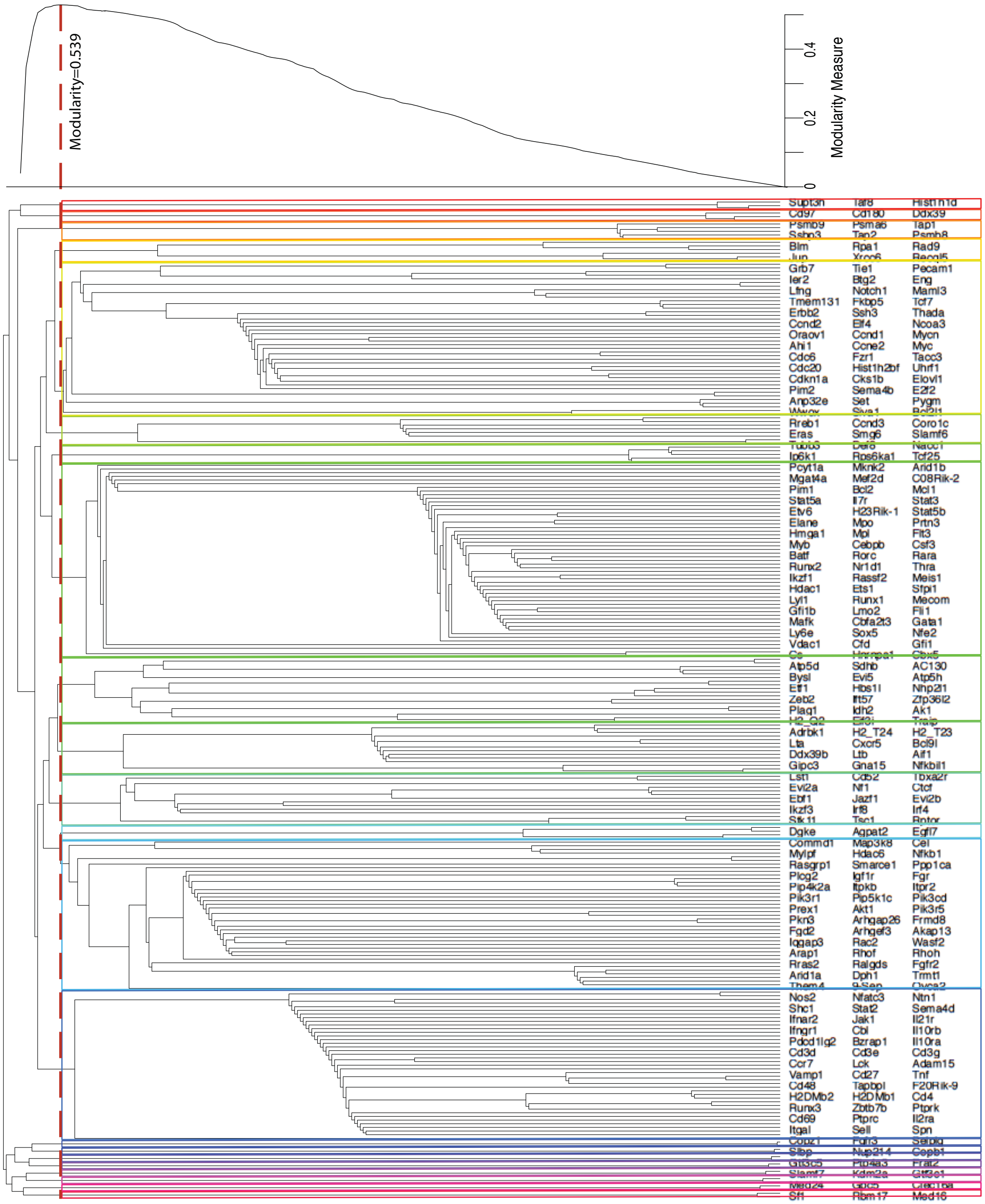

Figure S6: The dendrogram extracted by the shortest-path betweenness clustering technique to divide ReMIC genes into densely connected subnetworks at the small-scale ($\beta = 0.005$). The vertical height of the joint points indicate the order in which the joints take place. The root of the dendrogram is the original network and the leaves are individual genes.The best cut-level of this dendrogram is indicated by the dashed line which is the local maxima of the modularity measure. ReMIC gene clusters are indicated in colors. The dendrogram is visualized using igraph R library [1].

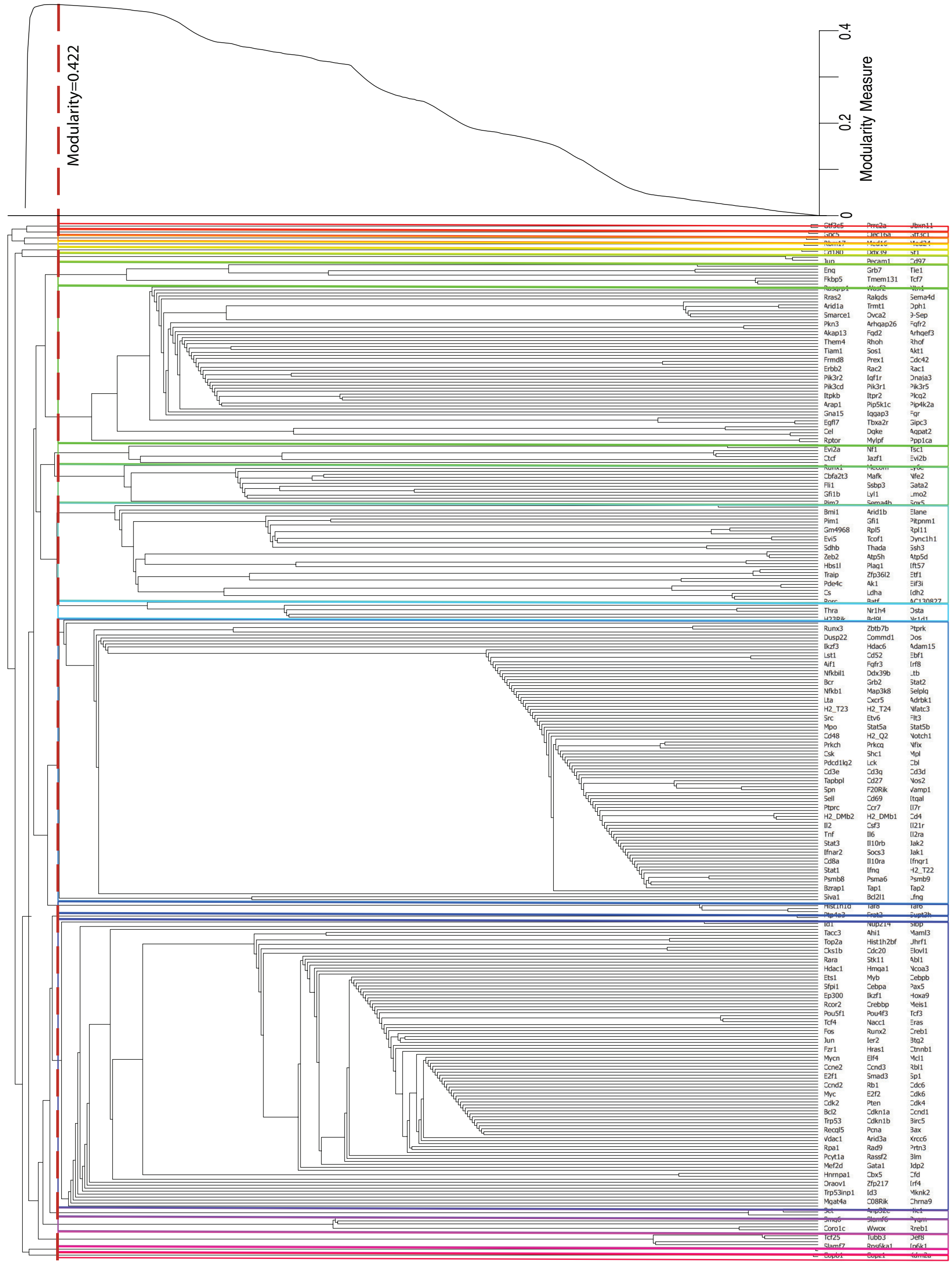

Figure S7: The dendrogram extracted by the shortest-path betweenness clustering technique to divide ReMIC genes into densely connected subnetworks at the small-scale ($\beta = 0.01$). The vertical height of the joint points indicate the order in which the joints take place. The root of the dendrogram is the original network and the leaves are individual genes.The best cut-level of this dendrogram is indicated by the dashed line which is the local maxima of the modularity measure. ReMIC gene clusters are indicated in colors. The dendrogram is visualized using igraph R library [1].

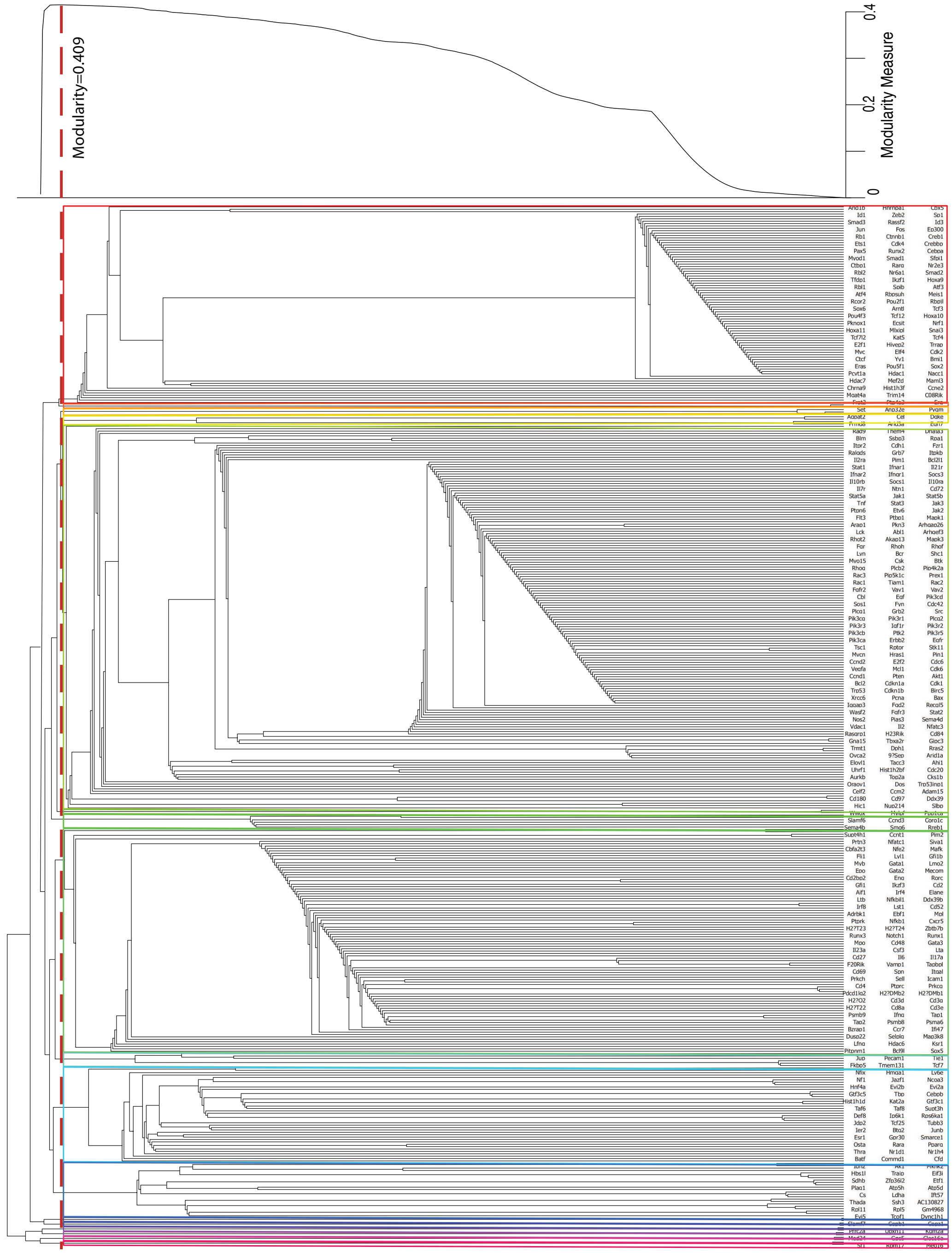

Figure S8: The dendrogram extracted by the shortest-path betweenness clustering technique to divide ReMIC genes into densely connected subnetworks at the medium-scale ($\beta = 0.015$). The vertical height of the joint points indicate the order in which the joints take place. The root of the dendrogram is the original network and the leaves are individual genes.The best cut-level of this dendrogram is indicated by the dashed line which is the local maxima of the modularity measure. ReMIC gene clusters are indicated in colors. The dendrogram is visualized using igraph R library [1].

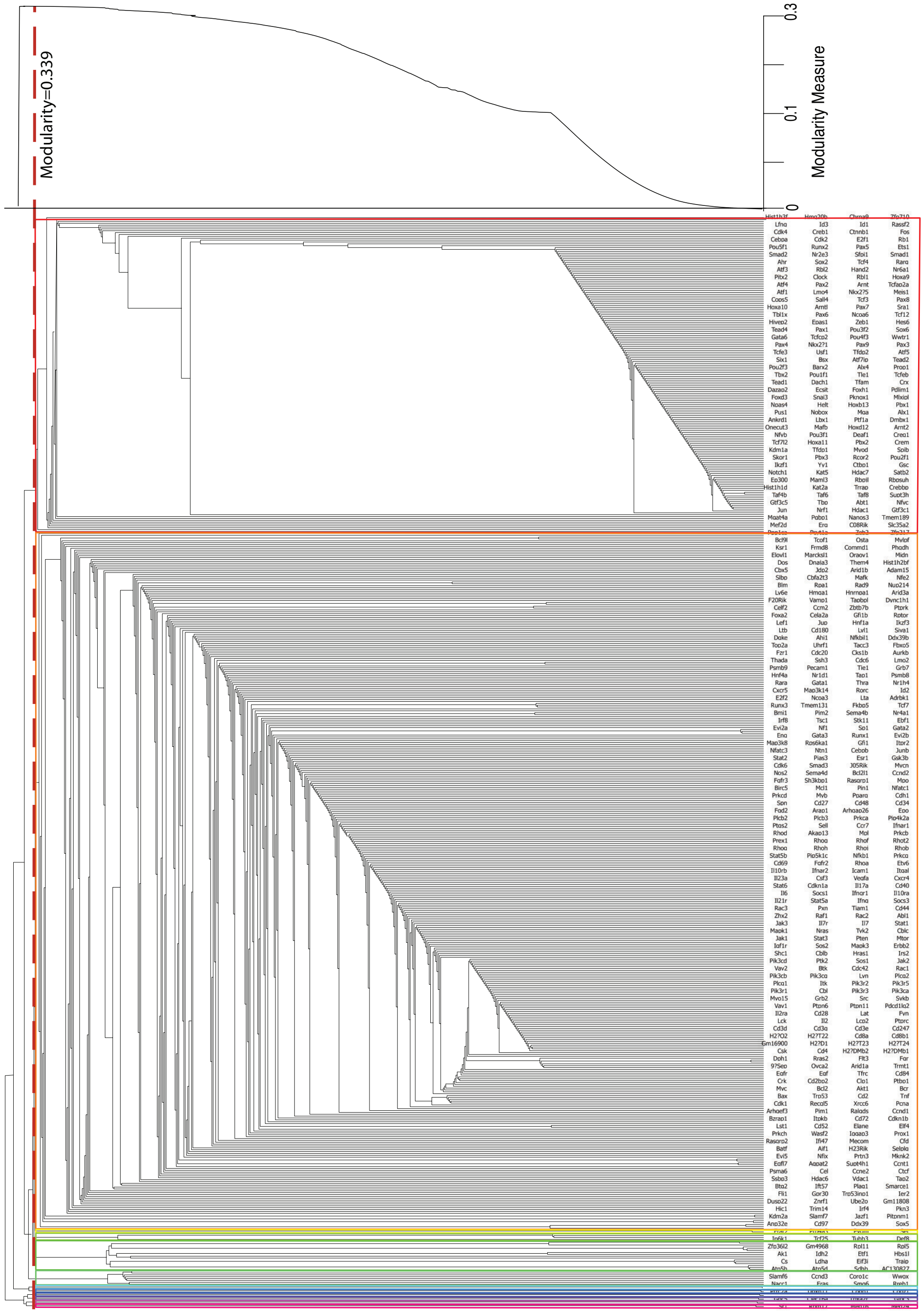

Figure S9: The dendrogram extracted by the shortest-path betweenness clustering technique to divide ReMIC genes into densely connected subnetworks at the medium-scale ($\beta = 0.02$). The vertical height of the joint points indicate the order in which the joints take place. The root of the dendrogram is the original network and the leaves are individual genes.The best cut-level of this dendrogram is indicated by the dashed line which is the local maxima of the modularity measure. ReMIC gene clusters are indicated in colors. The dendrogram is visualized using igraph R library [1].

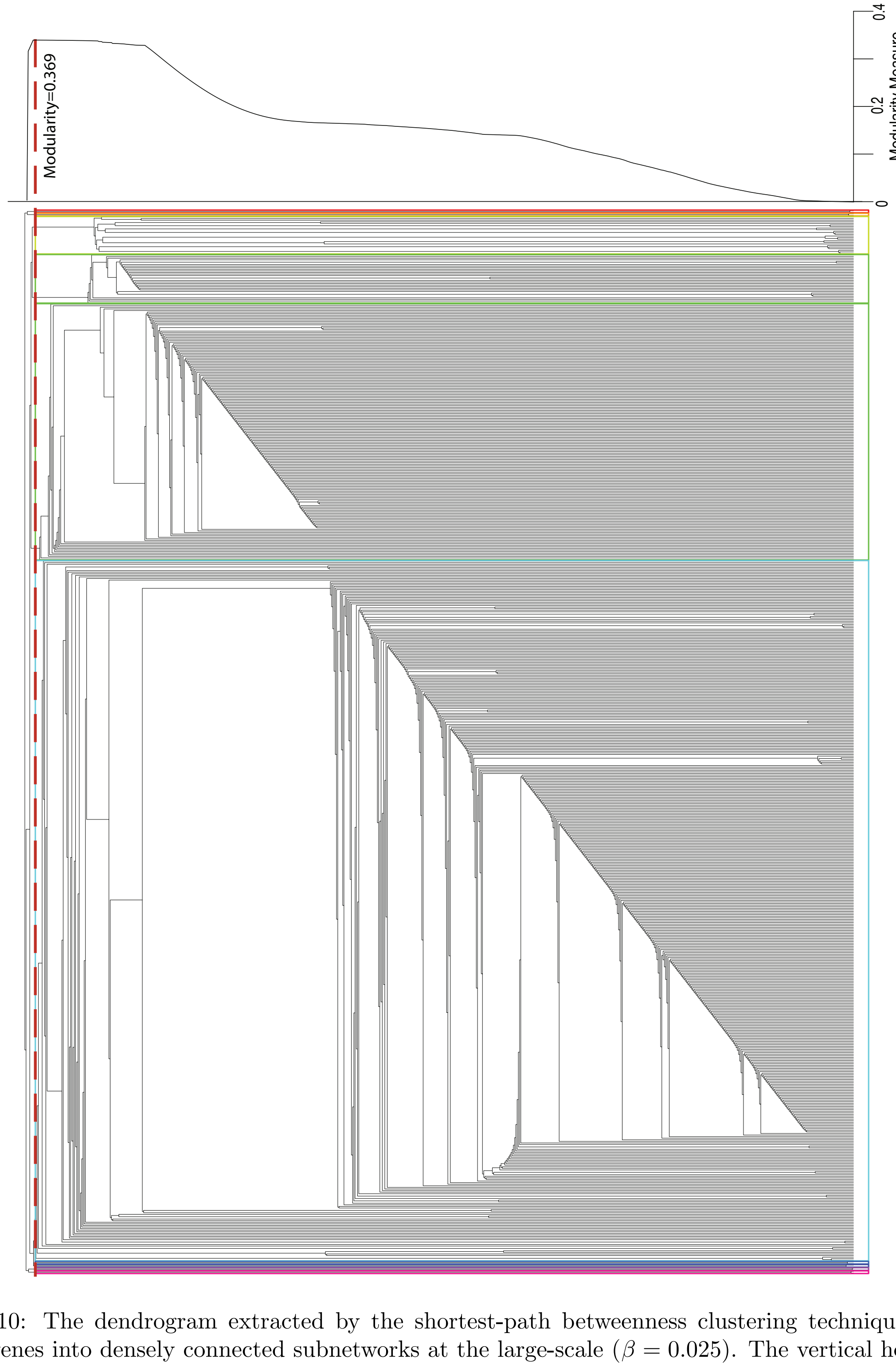

Figure S10: The dendrogram extracted by the shortest-path betweenness clustering technique to divide ReMIC genes into densely connected subnetworks at the large-scale ($\beta = 0.025$). The vertical height of the joint points indicate the order in which the joints take place. The root of the dendrogram is the original network and the leaves are individual genes.The best cut-level of this dendrogram is indicated by the dashed line which is the local maxima of the modularity measure. ReMIC gene clusters are indicated in colors. The dendrogram is visualized using igraph R library [1].

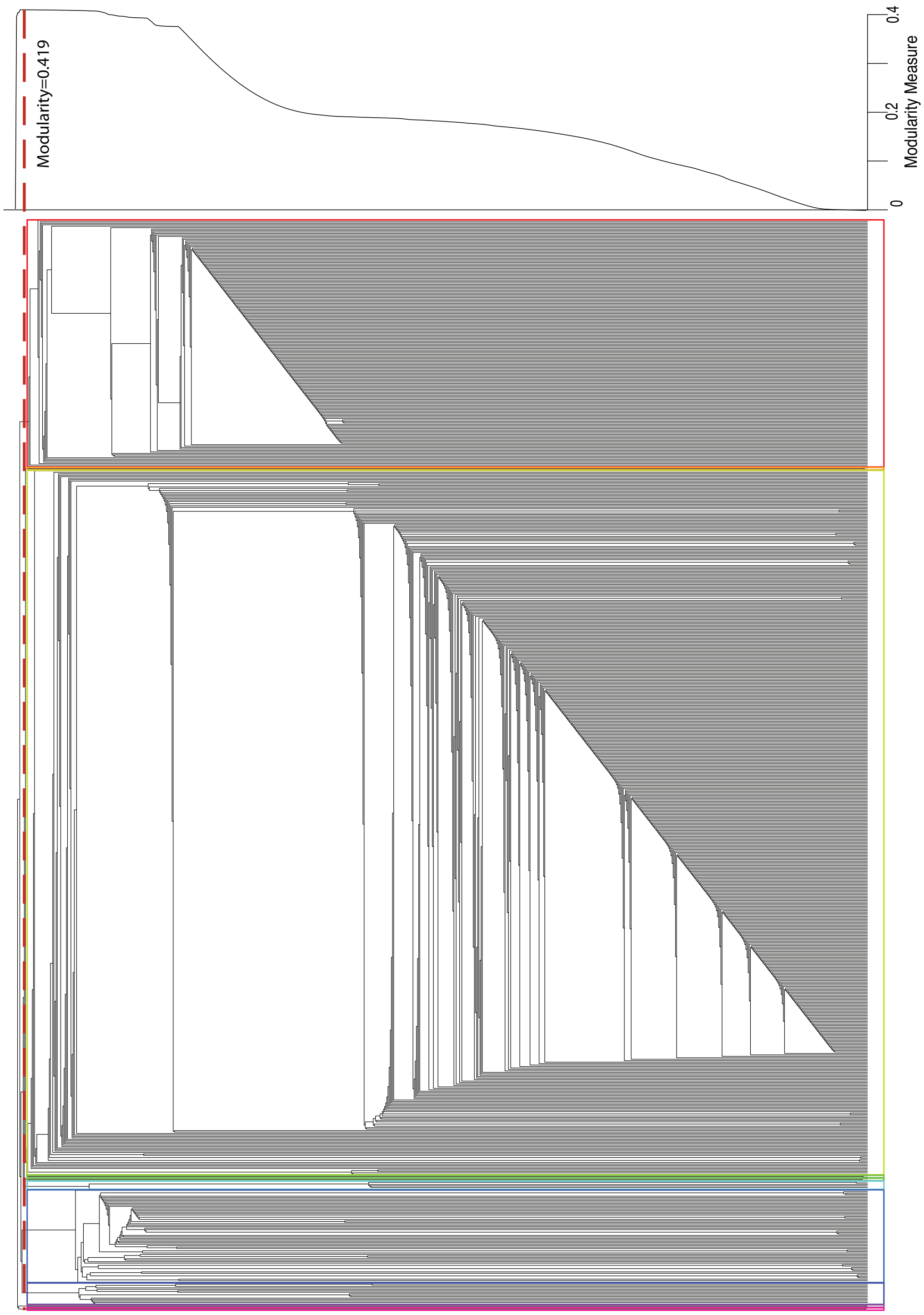

Figure S11: The dendrogram extracted by the shortest-path betweenness clustering technique to divide ReMIC genes into densely connected subnetworks at the large-scale ($\beta = 0.03$). The vertical height of the joint points indicate the order in which the joints take place. The root of the dendrogram is the original network and the leaves are individual genes.The best cut-level of this dendrogram is indicated by the dashed line which is the local maxima of the modularity measure. ReMIC gene clusters are indicated in colors. The dendrogram is visualized using igraph R library [1].

References

1. Csardi G, Nepusz T: **The igraph software package for complex network research**. *Int. J. Complex Syst* 2006, :1695.
